# Effects of Macrolide Treatment during the Hospitalization of Children with Childhood Wheezing Disease: A Systematic Review and Meta-Analysis

**DOI:** 10.3390/jcm7110432

**Published:** 2018-11-09

**Authors:** Chien-Yu Lin, Tzu-Lin Yeh, Shu-Jung Liu, Hsin-Hui Lin, Yu-Jyun Cheng, Hua-His Hung, Mu-Chieh Tsai, Jui-Ming Liu, Wei-Te Lei

**Affiliations:** 1 Department of Pediatrics, Hsinchu MacKay Memorial Hospital, Hsinchu 30071, Taiwan; mmhped.lin@gmail.com (C.-Y.L.); 4569@mmh.org.tw (Y.-J.C.); shi03312003@gmail.com (H.-H.H.); alimu0515@gmail.com (M.-C.T.); 2 Department of Family Medicine, Hsinchu MacKay Memorial Hospital, Hsinchu 30071, Taiwan; 5767@mmh.org.tw; 3 Institue of Epidemiology and Preventive Medicine, National Taiwan University, Taipei 10055, Taiwan; 4 Department of Medical Library, MacKay Memorial Hospital, Tamsui Branch, New Taipei City 25160, Taiwan; sjliu@mmh.org.tw; 5 Department of Family Medicine, Taipei MacKay Memorial Hospital, Taipei 10449, Taiwan; huilin0205@gmail.com; 6 Division of Urology, Department of Surgery, Taoyuan General Hospital, Ministry of Health and Welfare, Taoyuan 33004, Taiwan; mento1218@gmail.com; 7 Graduate Institute of Life Sciences, National Defense Medical Center, Taipei 11490, Taiwan; 8 Graduate Institue of Clinical Medical Sciences, College of Medicine, Chang Gung University, Taoyuan City 33302, Taiwan

**Keywords:** wheezing, asthma, bronchiolitis, macrolide, azithromycin, childhood wheezing disease

## Abstract

Children are susceptible to a variety of respiratory infections. Wheezing is a common sign presented by children with respiratory infections. Asthma, bronchiolitis, and bronchitis are common causes of childhood wheezing disease (CWD) and are regarded as overlapping disease spectra. Macrolides are common antimicrobial agents with anti-inflammatory effects. We conducted a comprehensive literature search and a systematic review of studies that investigated the influences of macrolide treatment on CWD. The primary outcomes were the impact of macrolides on hospitalization courses of patients with CWD. Data pertaining to the study population, macrolide treatment, hospital courses, and recurrences were analyzed. Twenty-three studies with a combined study population of 2210 patients were included in the systematic review. Any kind of benefit from macrolide treatment was observed in approximately two-thirds of the studies (15/23). Eight studies were included in the meta-analysis to investigate the influence of macrolides on the length of stay (LOS), duration of oxygen demand (DOD), symptoms and signs of respiratory distress, and re-admission rates. Although the benefits of macrolide treatment were reported in several of the studies, no significant differences in LOS, DOD, symptoms and signs of respiratory distress, or re-admission rates were observed in patients undergoing macrolide treatment. In conclusion, any kind of benefit of macrolide treatment was observed in approximately two-thirds of the studies; however, no obvious benefits of macrolide treatment were observed in the hospitalization courses of children with CWD. The routine use of macrolides to improve the hospitalization course of children with CWD is not suggested.

## 1. Introduction

Respiratory tract infections are common in children. Wheezing is a common sign of a respiratory disease [1]. It is a result of inflammation and narrowing of the airways and is common in children because of the immature nature of the anatomy of their respiratory tract and immune system. Asthma, bronchiolitis, and bronchitis are leading causes of childhood wheezing disease (CWD) [2]. Children with bronchiolitis have a significantly higher risk of subsequently developing asthma [3,4]. These diseases share many overlapping similarities as it relates to pathophysiology, clinical manifestations, treatment, and prognosis. They are regarded as a disease spectrum of different ages and stages. CWD is an important health issue with a huge disease burden worldwide [5]. In the United States, the prevalence of asthma was approximately 10% in 2010. Asthma was responsible for 10 hospital visits per 1000 children [6]. Although the prevalence of CWD varies in different areas, it remains an important health threat worldwide.

Antibiotic use is not beneficial in treating viral infections and should not be administered to patients with asthma, acute bronchiolitis, or bronchitis [7]. A macrolide is a polyketide antimicrobial agent. Examples include erythromycin, clarithromycin, azithromycin, telithromycin, and fidaxomicin. They are effective against atypical infections, such as *Mycoplasma pneumoniae*, *Chlamydophila pneumoniae*, and pertussis [8,9]. In addition to their antimicrobial effects against atypical pathogens, the anti-inflammatory properties of macrolides have been recognized. Various immunological reactions are affected by macrolides [10,11,12]. Decreases in the number of neutrophils, interleukin (IL)-8, IL-6, IL-1beta, tumor necrosis factor (TNF)-alpha, eosinophilic cationic protein, and matrix metalloproteinase 9 were observed in patients undergoing macrolide treatment [12]. Macrolides also cause a decrease in the type 2 T helper (Th2) cell cytokines (IL-4, IL-5, IL-6) and have potential immunomodulatory roles in treating chronic inflammatory diseases [12].

Inflammatory reactions play important roles in the pathophysiology of CWD. In patients with asthma, Th2 dominates responses, and immune-mediated cytokine cascades are known to play crucial roles [13]. Cytokine alterations were also observed in patients with bronchiolitis and bronchitis [14]. The immunomodulatory effects of macrolides on Th2 cell cytokines may decrease the inflammatory reactions of CWD [15]. Furthermore, Mycoplasma infection is a common causative and triggering pathogen of CWD [16,17]. *Mycoplasma pneumoniae* was found to account for 3.3–50% of acute exacerbations of asthma [17]. Therefore, it is reasonable to treat CWD patients with macrolides. Some studies have been conducted to explore the influence of treatment with macrolides on CWD patients but the findings were inconsistent. Kew et al. performed a systematic review to investigate the effects of macrolides on wheezing diseases in both adults and children. They found no significant differences in the hospital courses between the macrolide-treated group and the control group [15]. However, the immune responses and physiological properties of adults and children are different and the potential benefits of macrolides in treating CWD in children remain unclear. Therefore, we conducted this systematic review and meta-analysis to evaluate the impact of macrolides on CWD, and we focused on the influence of macrolides on the hospitalization courses.

## 2. Materials and Methods

### 2.1. Study Design and Study Selection

This study was approved by the Ethics Committee of the MacKay Memorial Hospital, Taiwan (registry no.: 16MMHIS035e). Our systematic review and meta-analysis were conducted in accordance with the Preferred Reporting Items for Systematic Review and Meta-Analysis Protocols [18]. The key terms used for the literature search were “asthma”, “bronchiolitis”, “bronchitis”, “wheezing”, “macrolide”, “erythromycin”, “clarithromycin”, “azithromycin”, and “telithromycin”. Keywords were combined using Boolean searches, and the searches were performed using keywords, Boolean operators, and MeSH descriptors. The detailed search strategy is shown in Table S1. We performed a systematic literature search in the following online databases: Embase; PubMed; the Cochrane Library; and the Cumulative Index to Nursing and Allied Health (CINAHL). All studies published as of August 2018 were eligible for inclusion. The Cochrane Collaboration Central Register of Controlled Clinical Trials, Cochrane Systematic Reviews, and ClinicalTrials.gov databases were manually searched for additional references. Two authors (S.-J.L. and T.-L.Y.) conducted the searches independently, and disagreements were resolved through discussion with the third author (C.-Y.L.).

After the initial search, two independent reviewers (W.-T.L. and C.-Y.L.) assessed the eligibility of each paper. The inclusion criteria were (1) inclusion of a control group in the study design; (2) administration of macrolides in one group; (3) children with a diagnosis of CWD; and (4) reporting of at least one treatment outcome of hospitalization. We excluded the following: (1) articles irrelevant to the topic; (2) duplicate publications; (3) trials with a crossover study design; (4) animal studies and studies conducted in adults; and (5) case reports and studies that did not include a control group.

### 2.2. Data Extraction and Quality Assessment

Two authors (W.-T.L. and C.-Y.L.) independently evaluated the quality of all eligible articles using the Cochrane Review risk of bias assessment tool. The adequacy of randomization, allocation concealment, blinding methods, implementation of the intent-to-treat analysis, dropout rate, complete outcome data, selective data reporting, and other potential biases were assessed for each publication.

The articles were scrutinized, and data pertaining to the following variables were extracted: study population; macrolide regimen, dosage, and duration; length of stay (LOS); duration of oxygen demand (DOD); chest indrawing or recession; crepitation, rhonchi, or crackles; cough; and admission rates. Discrepancies between the two independent evaluations for potential articles were resolved through discussion and consensus. Various kinds of influences have been reported in patients with macrolide treatment, including alterations in clinical courses, reduction of steroid or bronchodilator use, improvement of pulmonary function tests, laboratory tests and cytokine alterations, and changes of bacterial profiles. We focused on the influences of macrolides on the hospitalization courses in patients with CWD; therefore, the primary outcome of our study was LOS. The secondary outcomes were clinical symptoms and signs of respiratory distress and admission rates after treatment.

### 2.3. Data Synthesis and Analysis

Details of hospitalization from all the studies were extracted, analyzed, and compared to determine differences in the influences of macrolides on hospitalization courses. Because of the significant (and expected) heterogeneity among the studies, a random effects model was employed [19]. The results are presented as point estimates with 95% confidence intervals (CIs). The heterogeneity across studies was tested using *I*-square and Cochran’s *Q* tests. A *p* value of <0.10 for the chi-squared test of the *Q* statistic or an *I*-square >50% was considered indicative of statistically significant heterogeneity [20]. A sensitivity analysis was performed by repeating the analysis after sequential exclusion of one study at a time, to observe the effect on the overall results. Potential publication bias was assessed by observing the symmetry of funnel plots and by using Egger’s test [21]. Comprehensive Meta-analysis software (version 3.0, Biostat, Englewood, NJ, USA) was used for our analyses. 

## 3. Results

### 3.1. Description of Studies and Quality Assessment

**Figure 1:**
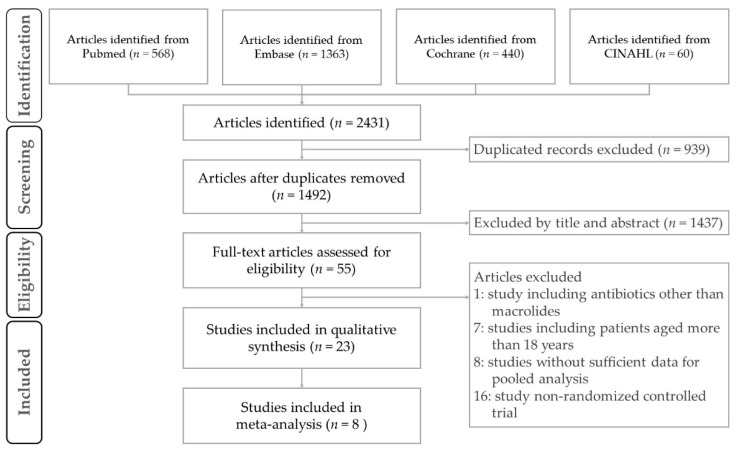
Schematic illustration of the literature search and the study selection criteria. CINAH, the Cumulative Index to Nursing and Allied Health.

A flowchart schematic illustration of the literature search and study selection criteria is presented in Figure 1. Finally, 23 publications were included in our qualitative synthesis and critical review (Table 1) [22,23,24,25,26,27,28,29,30,31,32,33,34,35,36,37,38,39,40,41,42,43,44]. Of these studies, 8 were conducted in the USA, and 14 studies investigated children younger than 5 years old. Azithromycin was used in most trials (13/23), while clarithromycin was used in 6 trials. Exclusion of patients with obvious bacterial infection, pneumonia, or recent antibiotics use was reported in 12 studies. Detection of causative pathogens was reported in 15 studies, and respiratory syncytial virus (RSV) was the most detected pathogen, appearing in 8 studies [26,31,32,36,37,38,42,44]. Evidence of *Mycoplasma pneumoniae* or *Chlamydophila pneumoniae* infection was reported in 5 studies with a range of 0–53% [24,28,30,33,41]. The treatment dosage and duration differed in different studies and concomitant inhaled corticosteroid use was reported in approximately one-third of the studies (9/23). In total, 2210 patients were enrolled in these studies. Any kind of benefit of macrolide treatment was observed in approximately two-thirds of the studies (15/23), including improvement of hospitalization courses, reduction of steroid or bronchodilator use, pulmonary function tests, laboratory tests, cytokine alterations, or bacterial profiles. Most of the included studies had a low potential for bias, as shown by our quality assessment using the Cochrane assessment tool. The detailed quality assessment of each included study is shown in Table S2. 

### 3.2. Data Synthesis and Meta-Analysis

Although any kind of benefit of macrolide treatment was observed in approximately two-thirds of the studies in our systematic review, we focused on the influences of macrolides on the hospitalization courses. Data pertaining to hospitalization courses including LOS, oxygen demand, and symptoms and signs of respiratory distress were extracted for further meta-analysis. Studies with different outcome measurements were excluded, such as steroid use, pulmonary function tests, laboratory tests, cytokine alterations, or bacterial profiles. Ultimately, eight studies—with a combined study population of 1357 patients—were included in our meta-analysis [26,27,29,31,32,35,37,38]. Two publications with the same study population were regarded as one trial [36,37]. Detailed data pertaining to our specific outcomes regarding LOS were not available for 14 studies; therefore, these studies were not eligible for the meta-analysis.

**Figure 2:**
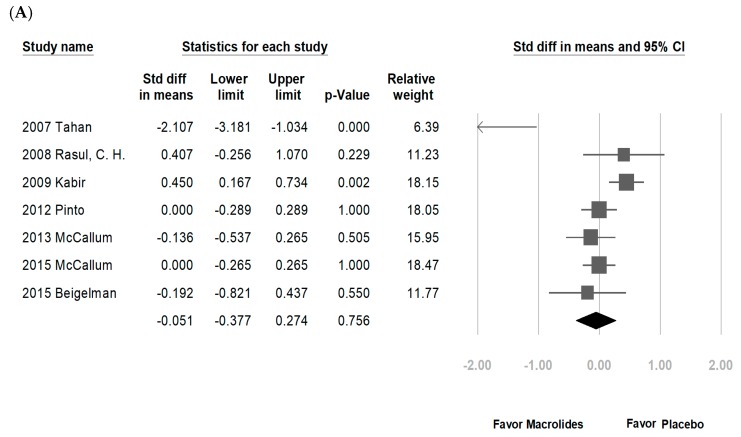

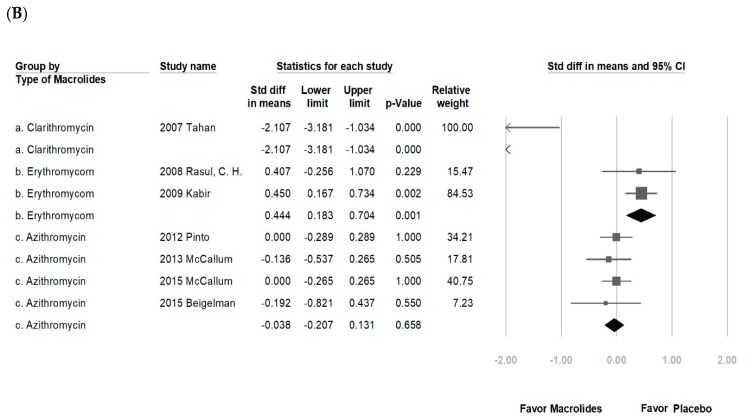
Forrest plot of the LOS in the macrolide-treated and placebo groups. (**A**) Overall meta-analysis; (**B**) subgroup analysis by macrolide category. CI: confidence interval; Std diff: standardized difference.

**Figure 3:**
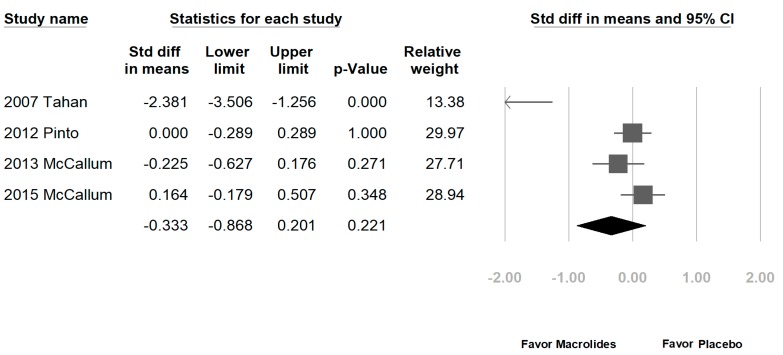
Forrest plot of the duration of oxygen demand (DOD) in the macrolide-treated and placebo groups.

**Figure 4:**
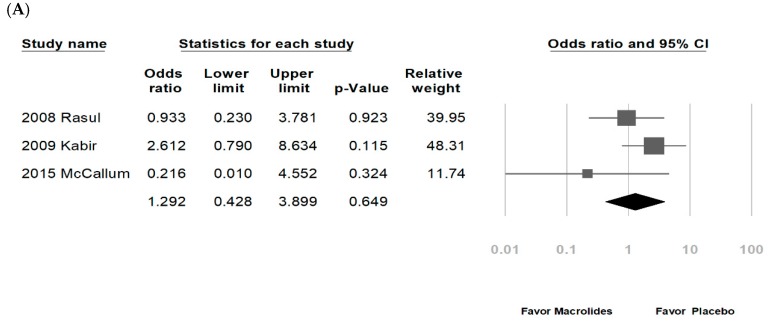

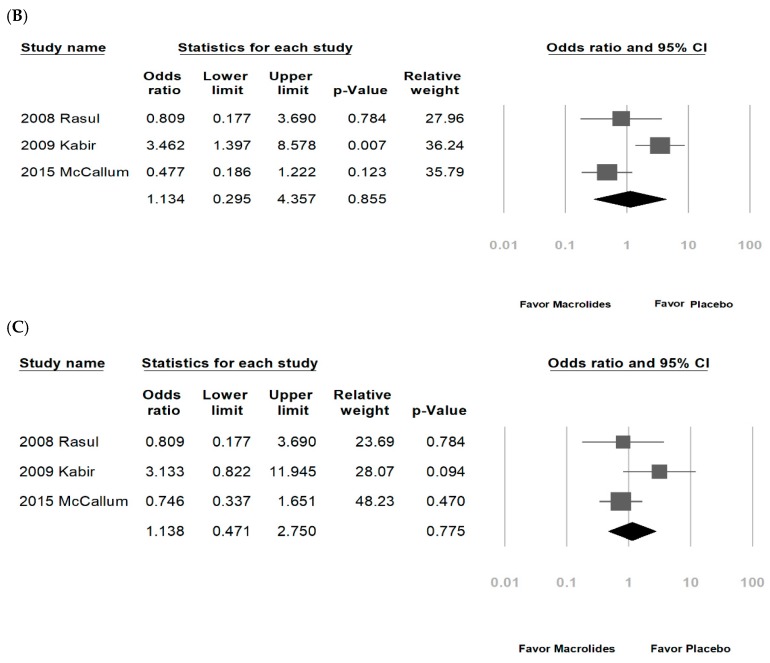
Forrest plot of the symptoms and signs of respiratory distress in the macrolide-treated and placebo groups. (**A**) Chest indrawing/recession; (**B**) crepitation, rhonchi, and crackles; (**C**) cough.

**Figure 5:**
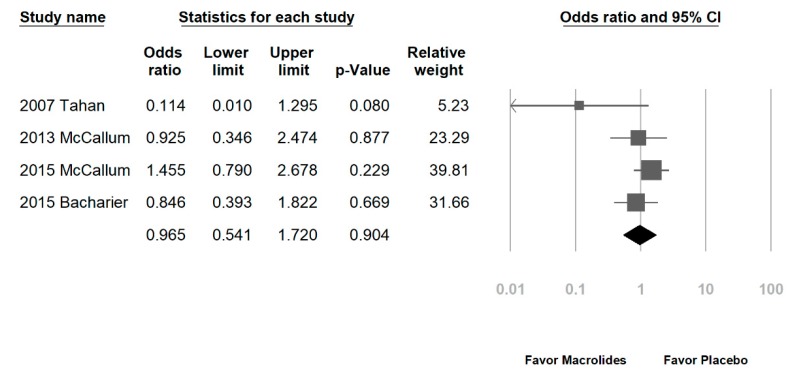
Forrest plot of the re-admission rates after this event in the macrolide-treated and placebo groups.

Among the eight enrolled studies, five were hospitalized patients with bronchiolitis, two were hospitalized patients with RSV bronchiolitis, and one was recurrent wheezing. No significant differences were noted regarding LOS in the macrolide treatment group (−0.051 days, range: −0.377 to 0.274 days, *p* = 0.756, *I*^2^ = 76.8%, Figure 2A). A subgroup analysis by macrolide category showed similar results, except for a longer LOS in the erythromycin-treated group (erythromycin: 0.444 days, range: 0.183–0.704 days, *p* = 0.001, *I*^2^ = 0%; azithromycin: −0.038 days, range: −0.207 to 0.131 days, *p* = 0.658, *I*^2^ = 0%; Figure 2B). Another subgroup analysis by disease category also showed no significant differences (figure not shown). The DOD was similar in both groups (−0.333 days, range: −0.868 to 0.201 days, *p* = 0.221, *I*^2^ = 84%, Figure 3). No significant differences in chest indrawing/recession were observed between the two groups (OR: 1.292, 95% CI: 0.428–3.899, *p* = 0.649, *I*^2^ = 29%, Figure 4A). The observed crepitation, rhonchi, and crackles were not significantly different (OR: 1.134, 95% CI: 0.295–4.357, *p* = 0.855, *I*^2^ = 78%, Figure 4B). Cough symptoms were similar in both groups (OR: 1.138, 95% CI: 0.471–2.75, *p* = 0.775, *I*^2^ = 41%, Figure 4C). Finally, there was no significant difference in re-admission rates after this event (OR: 0.965, 95% CI: 0.541–1.72, *p* = 0.904, *I*^2^ = 36%, Figure 5). The funnel plots were also assessed.

## 4. Discussion

The attractive antimicrobial properties against atypical pathogens and anti-inflammatory effects of macrolides may be beneficial in treating CWD, and our systematic review found that any kind of benefit from macrolide treatment was observed in approximately two-thirds of enrolled studies (15/23), including improvement in hospitalization course, steroid or bronchodilator use, pulmonary function, laboratory tests, cytokine alterations, or bacterial profiles. However, from the point of view of hospitalization course, no significant benefits in terms of LOS, DOD, symptoms and signs of respiratory distress, or re-admission rates were observed between macrolide-treated and control groups. Routine use of macrolides in treating patients with CWD to improve hospitalization course is therefore not recommended.

CWD may be triggered or aggravated by infectious pathogens [4,17,45,46]. In patients with previously diagnosed asthma, *Mycoplasma pneumoniae* was found to be the causative agent in 20% of those with acute exacerbations [16]. It accounted for 3.3–50% of acute exacerbations of asthma in other reports [17]. Pertussis was a factor in up to 21% of patients with a chronic cough in a Taiwanese population [47]. Macrolides are effective against these atypical pathogens and may improve the treatment course of CWD [48]. In our systematic review, exclusion of patients with obvious infection, pneumonia, or recent antibiotics use was reported in 12 studies, and detection of pathogens was performed in 15 studies. Evidence of *Mycoplasma pneumoniae* or *Chlamydophila pneumoniae* infection was reported in five studies with a broad range of 0–53%, and these five studies were not included in our meta-analysis. We did not have adequate evidence to confirm the influence of the antimicrobial properties of macrolides. Furthermore, the pathophysiology of CWD is complex, and the Th1/Th2 hypothesis is an important theory of asthma [13,49]. Th2 predominant reactions and cytokines were cardinal findings observed in patients with asthma [14,50,51]. Elevation of IL-4, IL-5, IL-9, and IL-13 was noted in asthma cytokine alterations [51]. In contrast, macrolides are found to inhibit Th2 cytokines, such as IL-4 and IL-5, and modulate extracellular signal-regulated kinase 1/2 and transcription factors in patients undergoing macrolide treatment [10,11,12]. Increasing evidence of the immune-modulatory effects of macrolides has been observed, and macrolides have been applied to treat some chronic inflammatory diseases [9,52]. Because of their antimicrobial and anti-inflammatory effects, the application of macrolides may be promising for the treatment of CWD [53]. However, no definite conclusion was made in previous studies. Our systematic review and meta-analysis concluded that although benefits of macrolides were reported in several studies, the evidence of the beneficial effects of macrolides in the treatment of CWD was not statistically significant regarding the LOS, DOD, symptoms and signs of respiratory distress, and re-admission rates. Routine use of macrolides in treating patients with CWD to improve hospitalization courses is therefore not supported by the current evidence.

Macrolides have both antimicrobial and anti-inflammatory effects, but increasing antimicrobial resistance (AMR) may affect the potential benefits of macrolides [54]. AMR is an important health threat worldwide and is associated with higher rates of treatment failure and poor prognosis. Recent studies have also noted increasing AMR of atypical pathogens. The resistance of *Mycoplasma pneumoniae* to macrolides was reported to be approximately 8.2% in the United States and up to 90% in some Asian countries [54,55,56]. In our systematic review, benefits of macrolides were observed in all reviewed studies published before 2007 (Table 1). Increasing AMR may contribute to the conflicting results of studies after 2007. Furthermore, vaccination may change the distribution of causative or trigger pathogens of CWD. Widespread vaccination against pertussis has been implemented in many countries for several decades. Although the effectiveness of current vaccination is not satisfactory, a dramatic decline in infections with pertussis was found in the last two decades [57]. Changes in the epidemiology of atypical pathogens may affect the contributing causative pathogens of CWD and decrease the potential benefits of treating CWD with macrolides.

RSV infection is common and severe in young infants with bronchiolitis [58]. Airway inflammation and hyper-responsiveness were found in patients with RSV infection. Macrolides may inhibit the inflammatory processes and improve the clinical course [59]. In our systematic review, RSV was found to be the most prevalent pathogen in eight studies. Three studies investigating patients with RSV bronchiolitis who were subsequently treated with macrolides were identified in our systematic review [26,36,37]. Benefits of macrolide use were reported in two studies [26,37]. Interestingly, two proof-of-concept studies with the same study population were conducted by Dr. Beigelman [36,37]. They found that macrolides could decrease the nasal lavage fluid levels of IL-8 and prolong the time to a third wheezing episode [37]. However, the RSV viral load was not decreased by macrolide treatment [36]. The influences, detailed mechanisms, and long-term effects of macrolide treatment require further studies.

Our study had some limitations. First, much heterogeneity was observed with respect to the study design, study participants, enrolled diagnoses, macrolide treatment, and the outcomes. CWD comprises several similar overlapping diseases, but the detailed pathophysiology and disease phenotypes may be different. Among the eight enrolled studies, five were hospitalized patients with bronchiolitis, two were hospitalized patients with RSV bronchiolitis, and one was recurrent wheezing. Hospitalization was common in young infants with bronchiolitis but relatively uncommon in older children with asthma. We performed a subgroup analysis of different age groups and diagnoses, but no significant conclusions could be made. Detection of causative pathogens and reporting of atypical infections were not performed in every study, and the role of the antimicrobial properties of macrolides remained largely unclear. Currently, the importance of individualized health care is reinforced, and the responses to macrolide treatment may be different in different individuals. Our systematic review was consistent with this concept, and regular use of macrolides in patients with CWD to improve hospitalization course is therefore not recommended. Additionally, the choices of macrolides and treatment duration and dosage were not the same among studies. There was no consensus regarding the optimal macrolide or the dosage and duration; thus, further studies are warranted. Furthermore, laboratory data, including cytokine alterations and detection of causative pathogens, were not available in all studies. A comparison of cytokine alterations after macrolide treatment is valuable in evaluating the inflammatory properties of macrolides. An analysis of causative microorganisms will contribute to elucidating the antimicrobial effects of macrolides. Finally, we focused on the impact of macrolides on the hospitalization courses of children with CWD. The potential long-term benefits of macrolide treatment were not investigated. Further studies are required to clarify the overall effect of macrolide treatment.

## 5. Conclusions

Macrolides have both antimicrobial and anti-inflammatory effects, and any kind of benefit from macrolide treatment was observed in approximately two-thirds of enrolled studies in our systematic review. However, from the point of view of hospitalization courses, our study suggests that macrolide treatment is not associated with the LOS, DOD, symptoms and signs of respiratory distress, or re-admission rates of CWD based on the currently available data. Therefore, routine treatment with macrolides to improve the hospital courses of children with CWD is not recommended. However, the potential long-term benefits of macrolide adjuvant treatment remain largely unclear, and possible benefits of macrolide treatment may exist in specific individuals. Further studies are warranted to elucidate the influence of macrolides on individual patients.

## Figures and Tables

**Table 1:** Characteristics of enrolled trials investigating macrolide treatment for childhood wheezing disease (CWD).

Study Author, Year [Ref]	Country	Study Population (M%: F%)	Severity, Diagnosis	Exclude Bacterial Infection?	Detect Pathogens?	Age	Macrolide Used	Dose, Interval	Duration	Concomitant Medication	Outcome Measure	Benefits of Macrolide?
Ball, 1990 [22]	USA	15 (60:40)	Severe asthma	N	NR	8–18 years	Troleando-mycin	250 mg QD × 2 days the QOD	14 days	Methyl-prednisolone	Steroid dose reduction Symptoms scores morning plasma cortisol concentration, FEV1, FVC, TGV, methacholine PC_20_, eosinophil count after 2 weeks	Y; increase steroid dose reduction and decrease bronchial hyperresonsiveness to methacholine
Kamada, 1993 [23]	USA	18 (36:64)	Severe asthma	NR	NR	6–17 years	Troleando-mycin	250 mg QD or QOD	12 weeks	Prednisolone, bronchodilator, theophylline, ICS	Steroid dose reduction, need for extra prednisolone Symptoms scores PEFR, pre-bronchodilator FEV1, FEF25-75%, methacholine PC_20_, morning plasma cortisol concentration, urinary cortisol excretion, bone density	Y; increase steroid dose reduction
Fonseca-Aten, 2006 [24]	USA	43 (74:26)	Recurrent wheezing, ED	Y; Exclude patients with bacterial infection	Y; evidences of *M pneumoniae* or *C peumoniae*: 53%	4–17 years	Clarithromycin	15 mg/kg/day, BID	5 days	β2-agonist and/or ICS	Serum/nasopharyngeal aspirates: TNF-α, IFN-γ, IL-1β, IL-2, IL-4, IL-5, IL-6, IL-8, IL-10, GM-CSF, RANTES, eotaxin, MIP-1α, MIP-1β, MCP-1 *M pneumoniae*, *C pneumoniae* detection in nasopharyngeal swabs/serologic test Dyspnea, wheeze, cough, asthma score	Y; decrease nasopharyngeal cytokine levels
Piacentini, 2007 [25]	Italy	16 (75:25)	Hospitalized, asthma	Y; None with airway infection in the month before and during study	NR	13.9 years	Azithromycin	10 mg/kg QD for 3 consecutive days/week	8 weeks	ICS, β2-agonist as needed	FEV1, FVC, FEF25-75%, bronchial hyperresponsiveness (expressed as the dose-response slope of FEV1 fall after hypertonic saline inhalation, and induced sputum)	Y; decrease bronchial hyperresponsiveness and sputum neutrophil percentage
Tahan, 2007 [26] *	Turkey	21 (57:43)	Hospitalized, RSV bronchiolitis	NR	Y; RSV	≤7 months	Clarithromycin	15 mg/kg QD	3 weeks	β2-agonist	Primary outcome: LOS; duration of need for O_2_, IVF and β2-agonist Secondary Outcomes: changes in the IL-4, IL-8, IFN-γ levels, readmission rate	Y; decrease LOS, DOD
Rasul, 2008 [27] *	Bangladesh	60 (72:28)	Hospitalized, bronchiolitis	N	NR	0–2 years (80% below 6 months)	Erythromycin	NR	ND	O_2_	Progress of the symptoms after 72 hours, progress of the signs after 72 hours, outcomes of bronchiolitis	N; no statistically significant differences found
Strunk, 2008 [28]	USA	55 (58:42)	moderate to severe persistent asthma	NR	Y; no *M pneumoniae*, *C pneumoniae* was detected by PCR	6–18 years	Azithromycin	250 mg or 500 mg QD	ND	ICS	Primary outcome: time to inadequate asthma control *M pneumoniae*, *C pneumoniae* detection in nasal washes: PCR assays	N; no differences in time to inadequate asthma control
Kabir, 2009 [29] *	Bangladesh	295 (73:27)	Hospitalized bronchiolitis	Y; Exclude patients with antibiotics use	NR	<24 months	Erythromycin	10 mg/kg/dose 6 hourly	ND	Inhaled bronchodilator, O_2_	Symptoms/signs which were graded on a two-point recovery scale of ‘rapid’ and ‘gradual’, indicating improvement within ‘four days’ and ‘beyond four days’, respectively	N; no differences among groups
Koutsoubari, 2012 [30]	Greece	40 (45:55)	Intermittent/mild persistent asthma	NR	Y; 18 rhinovirus, 3 adenovirus, 2 *M pneumoniae*, 2 parainfluenza, 1 RSV, and no *C pneumoniae*	6–14 years	Clarithromycin	15 mg/kg	3 weeks	ICS	Primary outcome: days without symptoms within subsequent 12 weeks Secondary outcome: symptom-free days after 1st AE, number/severity of periods with loss of control, time to 1st loss of control, PEFR variability, duration of the index episode, FEV1, Mean daily morning PEFR; RT-PCR in nasal wash samples	Y; increase symptom-free days and improve asthma control
Pinto, 2012 [31] *	Brazil	184 (60:40)	Hospitalized, bronchiolitis	Y; exclude Chlamydia spp or *Bordetella pertussis* respiratory infection	Y; RSV, influenza, and parainfluenza	≤2 months	Azithromycin	10 mg/kg/day	7 days	Antibiotics, Steroid, bronchodilator	Primary outcomes: LOS, duration of O_2_ Other variables: antibiotic use, broncho-dilators use, admission to the PICU, immunofluorescence for adenovirus, parainfluenza, influenza, RSV.	N; no differences in LOS, DOD, detected viruses
McCallum, 2013 [32] *	Australian/New Zealand	96 (68:32)	Hospitalized, bronchiolitis	Y; exclude patients with macrolide treatment or diagnoses of pneumonia	Y; RSV, rhinovirus, metapneumovirus, corona virus, and bacteria	≤18 months	Azithromycin	30 mg/kg	Single dose	Antibiotics	Primary endpoints: LOS, duration of O_2_ Other outcomes: any respiratory related readmissions in 6 months of discharge, identification of respiratory viruses and bacterial pathogens (RT-PCR/culture)	N; no differences in LOS, DOD, and re-admission rates
Chiong-Manaysay, 2014 [33]	Philippines	23	FEV1 <80% before treatment	NR	Y; 1 (4.8%) positive for *M pneumoniae*	Children	Clarithromycin	15 mg/kg/day bid	3 weeks	NR	Asthma Control Test questionnaires Spirometry (FVC, FEV1, FEV1/FVC, FEF25–75% and PEFR) prior medication and after the study period	Y; improved asthma control and FEV1
Youssef D, 2014 [34]	Greece	80 (44:36)	Persistent asthma	NR	NR	11.5 years	Clarithromycin	15 mg/kg bid	8 weeks	ICS, β2-agonist	FEV1 Eosinophils	N; significant decrease of neutrophils
Bacharier, 2015 [35] *	USA	443 (62:38)	recurrent, severe wheezing	Y; exclude patients received antibiotics within the past month for any indication	Y; viral pathogens were detected in 47% of children in the azithromycin group and 43%in the placebo group	12–71 months	Azithromycin	12 mg/kg/day	5 days	β2-agonist	Primary outcome: number of RTIs not progressing to a severe LRTI (prescription of oral corticosteroids) Secondary outcome: numbers of urgent care/ED visits, hospitalizations. Symptom scores, albuterol use, time to 2nd RTI	Y, lower risk to progress to severe LRTI
Beigelman, 2015 [36] *	USA	39 (59:41)	Hospitalized, RSV bronchiolitis	Y; treatment with any antibiotics within past 2 weeks (4 weeks for macrolide antibiotics)	Y; RSV	1–18 months	Azithromycin	10 mg/kg/day × 7 days then 5 mg/kg/day × 7 days	14 days	Antibiotic	Primary outcomes: serum and nasal lavage IL-8 levels, proportion of participants with ≥2 additional wheezing episodes after treatment Secondary outcomes: proportion of participants with ≥3 wheezing episodes, with diagnosed asthma, being-prescribed with ICS, the time to 2nd and 3rd episode, the number of, ED visits for respiratory symptoms	Y; decrease of nasal lavage IL-8 levels but not serum IL-8 levels. ≥2 additional wheezing episodes after treatment: not different
Beigelman, 2015 [37]	USA	39 (59:41)	Hospitalized, RSV bronchiolitis	Y; exclude patients with treatment with any antibiotics within past 2 weeks (4 weeks for macrolide antibiotics)	Y; RSV	1–18 months	Azithromycin	10 mg/kg/day × 7 days then 5mg/kg/day × 7 days	14 days	Antibiotic	RSV load in nasal lavage samples	N; azithromycin-treated group had lower RSV clearance
McCallum, 2015 [38] *	Australia/New Zealand	219 (62:38)	Hospitalized, bronchiolitis	Y; exclude patients received macrolides within last seven-days, or a primary pneumonia; non-macrolide antibiotics: 43%	Y; RSV (42%), rhinovirus (37%), adenovirus (7%), etc.	≤24 months	Azithromycin	30 mg/kg/dose weekly	3 weeks	Non-macrolide antibiotics	Primary endpoint: LOS, duration of O_2_, day 21 clinical review, 6 months readmission; Microbiology: Nasopharyngeal swabs for virus/bacteria (RT-PCR/culture)	Y; no differences of LOS, DOD and readmission. Nasopharyngeal bacteria were less common in azithromycin group
D’Azevedo Silveira, 2016 [39]	Brazil	91	Hospitalized, bronchiolitis	NR	NR	<12 months	Azithromycin	ND	7 days	NR	Wheezing and hospitalization in a follow up 1, 3 and 6 months	Y; readmission was not different but azithromycin group had lower recurrent wheezing
Stokholm, 2016 [40]	Denmark	72 (65:35)	recurrent asthma-like symptoms, troublesome lung symptoms ≥3 days	Y; exclude patients with signs of pneumonia	Y; any virus (43%), any bacteria (67%, *H influenzae*, *S pneumoniae*, *M catarrhalis*); not modify treatment effects	1–3 years	Azithromycin	10 mg/kg/day	3 days	ICS, Montelukast	Primary outcome: duration of episodes of troublesome lung symptoms Secondary outcomes: time from treatment to the next episode of troublesome lung symptoms, episodes that turned into severe AE, and the duration of β2 agonist use after treatment.	Y; azithromycin shortened the symptomatic period and the duration of β2 agonist use. Time to next episode was not different.
Wan, 2016 [41]	Taiwan	56 (63:37)	Mild persistent asthma	NR	Y; positive M *pneumoniae*: 58.3% for IgG and 41.7% for IgM in the study group, and 65.0% for IgG and 35.0% for IgM in the control group	5–16 years	Clarithromycin	5 mg/kg/day	4 weeks	ICS	Childhood asthma control test, FEV1, FEF25-75%, FeNO, total IgE, absolute eosinophil count, ECP level	Y; improve pulmonary function and decrease eosinophilic inflammation and disease severity
Zhou, 2016 [42]	USA	39 (59:41)	Hospitalized, RSV bronchiolitis	Y; exclude patients with treatment with any antibiotics within past 2 weeks (4 weeks for macrolide antibiotics)	Y; RSV and bacteria (*Moraxella*)	1–18 months	Azithromycin	10 mg/kg/day × 7 days then 5 mg/kg/day × 7 days	14 days	Antibiotic	Recurrent wheezing: assessed monthly over a year following the initial episode Microbiome sequencing => Changes in nasal lavage microbial communities	Y; the relative abundance of Moraxella decreased significantly
Mandhane, 2017 [43]	Canada	222 (72:28)	Wheezing, ED	Y; exclude patients with antibiotics use in the past 30 days	NR	12–60 months	Azithromycin	10 mg/kg/day at day 1 then 5 mg/kg/day × 4 days (day 2–5)	5 days	ICS, β2-agonist	Primary outcome: time (days) to respiratory symptoms resolution Secondary outcomes: the number of days children used a SABA during the 21 day follow-up, time to disease exacerbation during the following 6 months	N
Pinto, 2017 [44]	Brazil	83	Hospitalized, bronchiolitis	NR	Y; RSV	<12 months	Azithromycin	ND	7 days	NR	LOS, identification of respiratory viruses, recurrent wheezing/hospital readmission	Y; subsequent wheezing was significant reduced. The readmission rate was not different.

* studies included in meta-analysis. Abbreviations (in alphabetical order): AB: acute bronchiolitis, AE: acute exacerbation, AGE: acute gastroenteritis, BHR: bronchial hyper-responsiveness, BID: twice per day, C: control, *C pneumoniae: Chlamydophiliia pneumoniae,* DRS: dose-response slope, ECP: eosinophil cation protein, ED: emergency department, ELISA: enzyme-linked immunosorbent assay, F: female, FEV1: forced expiratory volume in one second, FVC: forced vital capacity, FeNO: exhaled nitric oxide levels, FEF25-75%: forced expiratory flow between 25% and 75% of vital capacity, ICS: inhaled corticosteroids, I: intervention, IVF: intravenous fluid, IL: interleukin, GM-CSF: granulocyte-macrophage colony stimulating factor, IFN: interferon, ICU: intensive care unit, LABA: long-acting inhaled β-agonists, LOS: length of stay, LRTI: lower respiratory tract infection, LTRA: leukotriene receptor antagonist, M: male, Methacholine PC_20_: concentration of methacholine required to induce a 20% decrease in FEV1, Nil: none, MIP: macrophage inflammatory protein, MCP: monocyte chemoattractant protein, *M pneumoniae: Mycoplasma pneumoniae,* N: No, NR: not reported, PEFR: peak expiratory flow rate, QD: every day, QOD: every other day, RSV: Respiratory syncytial virus, RTI: respiratory tract infection, RR: respiratory rate, RT-PCR: real-time polymerase chain reaction, SpO2: saturation of peripheral oxygen, SABA: short-acting beta agonist, SD: standard deviation, TGV: thoracic gas volume, Y: Yes.

## Supplementary Materials

The following are available online at http://www.mdpi.com/2077-0383/7/11/432/s1, Table S1: Detailed search strategy of our literature review, Table S2: Detailed quality assessment of each included study.
