# Supplementary material for: Effects of Macrolide Treatment during the Hospitalization of Children with Childhood Wheezing Disease: A Systematic Review and Meta-Analysis
Source: J Clin Med. 2018 Nov 9;7(11):432. doi: 10.3390/jcm7110432 (PMC6262331; doi:10.3390/jcm7110432)
Supplement: Supplementary file 1 [file jcm-07-00432-s001.zip › Table S2 COCHRANE RISK OF BIAS TOOL.docx]

Table S2. Risk of bias assessment of each included study^a^

| Study Validity Domains | Sequence generation | Allocation Concealment | Blinding of participants and personnel | Blinding of outcome assessment | Incomplete outcome data | Selective outcome reporting | Other sources  of bias |
| --- | --- | --- | --- | --- | --- | --- | --- |
| Ball 1990 | Unclear | Unclear | Low | Low | Low | Unclear | Low |
| Kamada 1993 | Low | Low | Low | Low | Low | Unclear | Unclear |
| Fonsecaaten 2006 | Unclear | Unclear | Unclear | Unclear | High | Low | Unclear |
| Piacentini 2007 | Low | Unclear | Low | Low | Unclear | Low | Unclear |
| Tahan 2007 | Low | Unclear | Low | Low | Low | Low | Unclear |
| Rasul 2008 | Low | Low | Low | Low | High | Low | Unclear |
| Strunk 2008 | Unclear | Unclear | Unclear | Unclear | Low | Low | Unclear |
| Kabir 2009 | Low | Unclear | Unclear | Unclear | Low | Low | Unclear |
| Koutsoubari 2012 | Low | High | High | High | Low | Low | Unclear |
| Pinto 2012 | Low | Unclear | Unclear | Unclear | Low | Low | Unclear |
| McCallum 2013 | Low | Low | Low | Low | Low | Low | Unclear |
| Chiong 2014 | Unclear | Unclear | Unclear | Unclear | Unclear | Unclear | Unclear |
| Youssef 2014 | Low | Unclear | Low | Low | Low | Unclear | Unclear |
| Bacharier 2015 | Low | Low | Low | Low | Low | Low | Unclear |
| Beigelman 2015(letter) | Low | Low | Low | Low | Low | Low | Unclear |
| Beigelman 2015 | Low | Low | Low | Low | Low | Low | Low |
| McCallum 2015 | Low | Low | Low | Low | Low | Low | Low |

Table S2. Risk of bias assessment of each included study (con’t)

| Study Validity Domains | Sequence generation | Allocation Concealment | Blinding of participants and personnel | Blinding of outcome assessment | Incomplete outcome data | Selective outcome reporting | Other sources  of bias |
| --- | --- | --- | --- | --- | --- | --- | --- |
| DAzeveda 2016 | Unclear | Unclear | Unclear | Unclear | Unclear | Unclear | Unclear |
| Stokholm 2016 | Low | Low | Low | Unclear | Low | Low | Low |
| Wan 2016 | Low | Low | Unclear | Unclear | Low | Low | Unclear |
| Zhou 2016 | Unclear | Unclear | Low | Low | Low | Low | Low |
| Mandhane 2017 | Low | Low | Low | Low | Unclear | Low | Low |
| Pinto 2017 | Unclear | Unclear | Unclear | Unclear | Unclear | Unclear | Unclear |

a. Each domain has been evaluated as being “High”, “Low”, or “Unclear” regarding the risk of bias following the guidelines of Cochrane
 Collaboration’s tool for assessing risk of bias, the thorough and original evaluation from is attached in the following pages

“Low” in all Domains would place a study at “Low Risk of Bias”; “High” in any of the Domains would place a study at “High Risk of Bias”; “Unclear” in any of the domains would place the study at “Unclear Risk of Bias”.
