# Supplementary material for: Effects of Macrolide Treatment during the Hospitalization of Children with Childhood Wheezing Disease: A Systematic Review and Meta-Analysis
Source: J Clin Med. 2018 Nov 9;7(11):432. doi: 10.3390/jcm7110432 (PMC6262331; doi:10.3390/jcm7110432)
Supplement: Supplementary file 1 [file jcm-07-00432-s001.zip › Table S1 Detailed searching strategy.docx]

Table S2. Detailed searching strategy of our systematic review

**PubMed**

| ((((((((((Asthma OR asthma*))) OR infantile asthma) OR chronic cough) OR ((Bronchitis, Chronic OR chronic bronchitis))) OR ((Respiratory Sounds OR wheezing OR wheez*))) OR (Bronchiolitis OR Bronchiolitides OR acute bronchiolitis)) OR (Bronchiolitis, Viral OR Viral Bronchiolitides))) in All Fields |
| --- |
| AND |
| ((((((Erythromycin OR T-Stat OR Erycette OR Erymax OR Ilotycin))) OR ((Clarithromycin OR TE-031 OR A-56268 OR Biaxin))) OR macrolide) OR ((Azithromycin OR Azythromycin OR Sumamed OR Toraseptol OR Vinzam OR CP-62993 OR CP 62993 OR CP62993 OR Zithromax OR Azitrocin OR Azadose OR Ultreon OR Zitromax OR Goxal OR Zentavion)))  in All Fields |
| Filters: Child: birth-18 years |

**Embase**

| (‘asthma’ OR ‘asthma’/exp OR asthma OR asthma*) OR (‘chronic bronchitis’/exp) OR (‘abnormal respiratory sound’/exp) OR (infantile NEAR/3 asthma) OR (chronic NEAR/3 cough) OR (chronic NEAR/3 bronchitis) OR (‘wheezing’/exp OR wheezing OR wheez*) OR (‘bronchiolitis’/exp) OR (‘viral bronchiolitis’/exp) OR ((viral AND bronchiolitides OR bronchiolitis OR bronchiolitides OR acute) AND bronchiolitis) OR ((viral NEAR/3 bronchiolitides) OR bronchiolitis OR bronchiolitides OR (acute NEAR/3 bronchiolitis)) |
| --- |
| AND |
| ('erythromycin'/exp OR erythromycin OR 't stat'/exp OR 't stat' OR 'erycette'/exp OR erycette OR 'erymax'/exp) OR (('azithromycin'/exp OR azithromycin OR 'azythromycin'/exp OR azythromycin OR 'sumamed'/exp OR sumamed OR toraseptol OR 'vinzam'/exp OR vinzam OR 'cp 62993'/exp OR 'cp 62993' OR cp) AND 62993 OR 'cp62993'/exp OR cp62993 OR 'zithromax'/exp OR zithromax OR 'azitrocin'/exp OR azitrocin OR 'azadose'/exp OR azadose OR 'ultreon'/exp OR ultreon OR 'zitromax'/exp OR zitromax OR goxal OR zentavion) OR ('clarithromycin'/exp OR clarithromycin OR 'te 031'/exp OR 'te 031' OR 'a 56268'/exp OR 'a 56268' OR 'biaxin'/exp OR biaxin) OR ('macrolides'/exp OR macrolides OR 'macrolide'/exp OR macrolide) |
| AND |
| ([adolescent]/lim OR [child]/lim OR [infant]/lim OR [newborn]/lim OR [preschool]/lim OR [school]/lim) |

**Cochrane**

| (MeSH descriptor: [Asthma] explode all trees) OR (MeSH descriptor: [Bronchitis, Chronic] explode all trees) OR (MeSH descriptor: [Respiratory Sounds] explode all trees) OR (MeSH descriptor: [Bronchiolitis] explode all trees) OR (MeSH descriptor: [Bronchiolitis, Viral] explode all trees) OR (Asthma or asthma* or infantile asthma or chronic cough or chronic bronchitis or Respiratory Sounds or wheezing or wheez*:ti,ab,kw (Word variations have been searched)  ) OR (Bronchiolitis or Bronchiolitides or acute bronchiolitis or Viral Bronchiolitides:ti,ab,kw (Word variations have been searched)) |
| --- |
| AND |
| (MeSH descriptor: [Erythromycin] explode all trees) OR (MeSH descriptor: [Azithromycin] explode all trees) OR (MeSH descriptor: [Clarithromycin] explode all trees) OR (MeSH descriptor: [Macrolides] explode all trees) OR (Erythromycin or T-Stat or Erycette or Erymax or Ilotycin:ti,ab,kw (Word variations have been searched)) OR (Azithromycin or Azythromycin or Sumamed or Toraseptol or Vinzam or CP-62993 or CP 62993 or CP62993 or Zithromax or Azitrocin or Azadose or Ultreon or Zitromax or Goxal or Zentavion:ti,ab,kw (Word variations have been searched)) OR (Clarithromycin or TE-031 or A-56268 or Biaxin:ti,ab,kw (Word variations have been searched)) OR (macrolides or macrolide*:ti,ab,kw (Word variations have been searched)) |

**CINAHL**

| (MH "Asthma+") OR ("chronic cough") OR (infantile asthma) OR (MH "Bronchitis, Chronic") OR  (MH "Respiratory Sounds") OR (MH "Bronchiolitis") OR (asthma* OR infantile asthma OR chronic cough OR chronic bronchitis OR Respiratory Sounds OR wheezing OR wheez* OR Bronchiolitis OR Bronchiolitides OR acute bronchiolitis OR viral bronchiolitis OR Viral Bronchiolitides) |
| --- |
| AND |
| ((MH "Erythromycin") OR "Erythromycin") OR (T-Stat OR Erycette OR Erymax OR Ilotycin) OR ((MH "Clarithromycin") OR "Clarithromycin") OR (TE-031 OR A-56268 OR Biaxin) OR ((MH "Antibiotics, Macrolide") OR "macrolide") OR ((MH "Azithromycin") OR "Azithromycin") OR (Sumamed OR Toraseptol OR Vinzam OR CP-62993 OR CP 62993 OR CP62993 OR Zithromax OR Azitrocin OR Azadose OR Ultreon OR Zitromax OR Goxal OR Zentavion) |
| Narrow by Subject Age: - all child |
